# Negatively charged, intrinsically disordered regions can accelerate target search by DNA-binding proteins

**DOI:** 10.1093/nar/gkad045

**Published:** 2023-02-13

**Authors:** Xi Wang, Lavi S Bigman, Harry M Greenblatt, Binhan Yu, Yaakov Levy, Junji Iwahara

**Affiliations:** Department of Biochemistry and Molecular Biology, Sealy Center for Structural Biology and Molecular Biophysics, University of Texas Medical Branch, Galveston, TX 77555-1068, USA; Department of Chemical and Structural Biology, Weizmann Institute of Science, Rehovot 76100, Israel; Department of Chemical and Structural Biology, Weizmann Institute of Science, Rehovot 76100, Israel; Department of Biochemistry and Molecular Biology, Sealy Center for Structural Biology and Molecular Biophysics, University of Texas Medical Branch, Galveston, TX 77555-1068, USA; Department of Chemical and Structural Biology, Weizmann Institute of Science, Rehovot 76100, Israel; Department of Biochemistry and Molecular Biology, Sealy Center for Structural Biology and Molecular Biophysics, University of Texas Medical Branch, Galveston, TX 77555-1068, USA

## Abstract

In eukaryotes, many DNA/RNA-binding proteins possess intrinsically disordered regions (IDRs) with large negative charge, some of which involve a consecutive sequence of aspartate (D) or glutamate (E) residues. We refer to them as D/E repeats. The functional role of D/E repeats is not well understood, though some of them are known to cause autoinhibition through intramolecular electrostatic interaction with functional domains. In this work, we investigated the impacts of D/E repeats on the target DNA search kinetics for the high-mobility group box 1 (HMGB1) protein and the artificial protein constructs of the Antp homeodomain fused with D/E repeats of varied lengths. Our experimental data showed that D/E repeats of particular lengths can accelerate the target association in the overwhelming presence of non-functional high-affinity ligands (‘decoys’). Our coarse-grained molecular dynamics (CGMD) simulations showed that the autoinhibited proteins can bind to DNA and transition into the uninhibited complex with DNA through an electrostatically driven induced-fit process. In conjunction with the CGMD simulations, our kinetic model can explain how D/E repeats can accelerate the target association process in the presence of decoys. This study illuminates an unprecedented role of the negatively charged IDRs in the target search process.

## Graphical Abstract

**Graphical Abstract:**
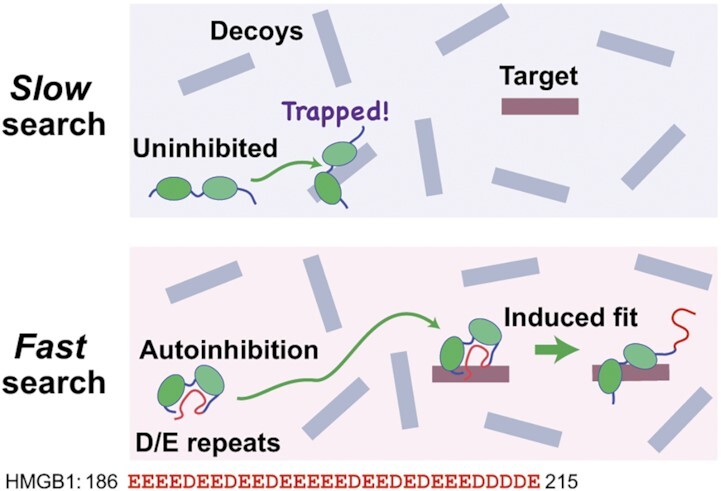
Negatively charged D/E repeats allow DNA-binding proteins to avoid distractions of nonfunctional high-affinity sites (‘decoys’) on DNA. This effect along with an induced-fit mechanism accelerates the target search process.

## INTRODUCTION

In eukaryotes, the majority of DNA-binding proteins such as transcription factors, histones, and other architectural proteins contain intrinsically disordered regions (IDRs) to a remarkable extent (1). Among human transcription factors, for example, IDRs occupy on average 50% of the protein sequence length (2). IDRs of DNA-binding proteins play important regulatory roles through protein-protein interactions, post-translational modifications, and liquid–liquid phase separation (3,4). Compared to foldable sequences, the sequences of IDRs are enriched in polar and charged amino acids (1). In some cases, highly negatively charged segments that contain only aspartate (D) or glutamate (E) residues are observed (5–20). We refer to them as ‘D/E repeats’.

**Figure 1.**
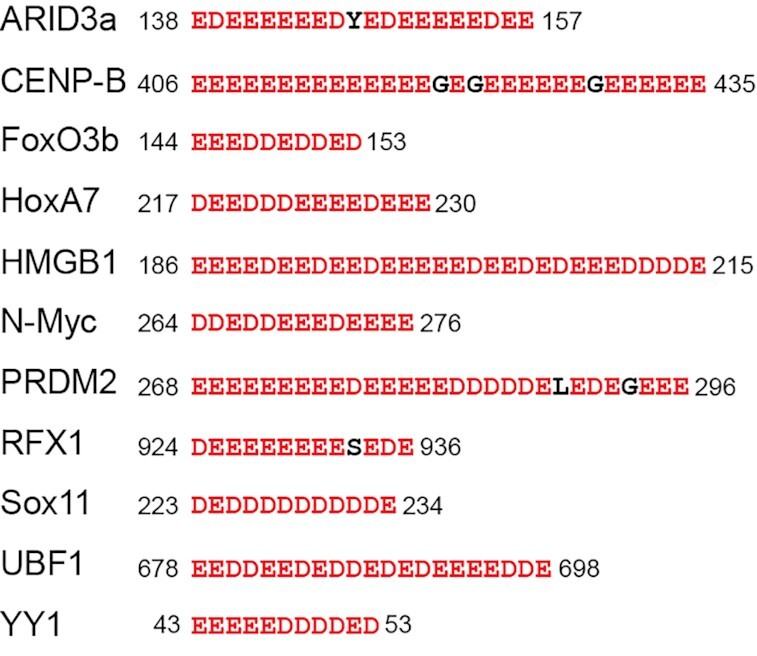
Examples of D/E repeats in human DNA-binding proteins. Autoinhibition by the D/E repeats has experimentally been shown for HMGB1, RFX1, Sox11 and UBF1 (5,7–11,13).

Bioinformatics studies revealed that D/E repeats are prevalent in the proteomes of eukaryotes (18,20). About a half of those proteins containing D/E repeats are DNA/RNA-binding proteins (20). Given the conformational flexibility and large negative charge of D/E repeats, it seems likely that D/E repeats can interact electrostatically with positively charged DNA-binding domains within the same polypeptide chain. Such interaction may cause autoinhibition that reduces apparent affinity for DNA. In fact, autoinhibition via negatively charged IDRs involving D/E repeats has been confirmed for some DNA-binding proteins, including HMGB1, RFX1, Sox11, and UBF1 (Figure 1) (7–9,11,13). However, the role of autoinhibition by D/E repeats is not well understood.

Autoinhibition is typically discussed in terms of a molecular switch between ‘on’ and ‘off’ states (21–23). In some cases, it is modulated by post-translational modifications such as phosphorylation (22,24–28). The autoinhibited state of a protein corresponds to the ‘off’ state of the molecular switch. However, such a description may be simplistic for DNA-binding proteins. While the functions of many DNA-binding proteins require binding to targets on DNA, numerous non-functional high-affinity sites (‘decoys’) can trap the proteins. For example, for transcription factors that recognize a particular sequence, the genome contains millions of natural decoys that can sequester the proteins (29–31). If the proteins remain uninhibited, abundant decoys may easily trap the proteins, impeding their association with the targets. Given this situation, autoinhibition of DNA-binding proteins may reduce the risk of the sequestration and thereby accelerate the protein-target association process in the overwhelming presence of decoys.

In this paper, we demonstrate that D/E repeats are well suited for such acceleration of target DNA search. To examine the impact of D/E repeats, we used the high-mobility group box 1 (HMGB1) protein and artificial constructs of the Antp homeodomain fused with D/E repeats tails (DERT) of varied lengths. HMGB1 contains 30-residue D/E repeats at the C-terminus. This protein also contains two DNA-binding domains that recognize atypical DNA such as cisplatin-modified DNA (32,33), Holliday junction (34,35), bulged DNA (36,37) and G-quadruplex (38,39). HMGB1 is a multi-function protein that play important roles in the nuclei as well as in extracellular space (40,41). In the nuclei, HMGB1 serves as a DNA chaperone and assists transcription factors, DNA-repair/recombination enzymes, and chromatin remodeling factors (40,42,43). Rapid access to distorted DNA is vital for HMGB1 (40). Based upon our previous studies on HMGB1 autoinhibition (13,44), we conducted kinetic experiments and found that the D/E repeats accelerate the target search process of HMGB1 in the overwhelming presence of decoys. Our data on the engineered proteins of the Antp homeodomain show that the acceleration effect of D/E repeats can be artificially implemented in other systems. Our coarse-grained molecular dynamics (CGMD) simulations and kinetic model provide great insights into the mechanism and conditions for the acceleration.

## MATERIALS AND METHODS

### HMGB1 and its variant

The human full-length HMGB1 and its Δ30 variant were expressed in *Escherichia coli* strain BL21(DE3) and purified using cation-exchange, anion-exchange, and size-exclusion chromatographic methods as described in our previous paper (13). The purified proteins were lyophilized and stored at -20°C until use. All cysteine residues were reduced to a thiol form by 5 mM DTT and the reduced state was confirmed by NMR as previously described.

### Antp homeodomain derivatives

The pET49a-derivative plasmid harboring a synthetic gene of the Antp HD-DERT16 protein at the NdeI/EcoRI sites was purchased from GenScript. The Antp HD-DERT16 was expressed in *E. coli* strain BL21(DE3) and purified through SP-FF cation-exchange, S-100 sephacryl size-exclusion, and Resource-Q anion exchange columns. The SP-FF column was equilibrated with 50 mM phosphate buffer (pH 7.5) and 0.1 M NaCl. The protein was eluted with a gradient of 0.1–2.0 M NaCl. The fractions containing the protein were concentrated to ∼10 ml and further purified by size exclusion chromatography equilibrated with a buffer of 50 mM Tris•HCl (pH 7.5), 1 mM EDTA, and 0.4 M NaCl. Fractions containing the Antp HD-DERT16 protein were combined, diluted two times with a buffer of 50 mM Tris•HCl (pH 7.5) and 1 mM EDTA, loaded onto a Resource-Q anion-exchange column, and eluted with a gradient of 0.2–1.5 M NaCl in 50 mM Tris•HCl (pH 7.5) and 1 mM EDTA. The plasmids for *E. coli* expression of the Antp HD–DERT11 protein, the Antp HD–DERT7 protein, and the control protein without DERT were generated from the plasmid for the Antp HD-DERT16 protein through mutagenesis using a QuikChange Lightning kit (Agilent). These proteins were purified in essentially the same manner as described above for Antp HD–DERT16 except that the Resource-Q anion-exchange column chromatography was replaced with the Resource-S cation-exchange with a gradient of 0–1.5 M NaCl in 50 mM Tris•HCl (pH 7.5) and 1 mM EDTA. Concentrations of individual proteins were measured using UV absorbance at 280 nm together with extinction coefficients predicted by the ProtParam tool (https://web.expasy.org/protparam/).

### Nucleic acids

Chemically synthesized DNA strands were purchased from Integrated DNA Technologies. Each strand was purified by anion-exchange chromatography. To prepare DNA duplexes, complementary strands were annealed, and excess single-stranded DNA was removed through anion-exchange chromatography. Concentrations of double-stranded DNA were measured using UV absorbance at 260 nm along with extinction coefficients calculated from the nucleotide sequences using the method of Tataurov et al (45).The fluorescence-labeled 20-bp DNA with a cisplatin modification were prepared as previously described (13). Yeast tRNA was purchased from Sigma-Aldrich (cat# 10109517001). To measure tRNA concentrations, UV absorbance at 260 nm was used along with an extinction coefficient of 7 × 10^5^ M^−1^cm^−1^, which was estimated from the average length and hypochromicity (46,47).

### Reagents

Chemicals used to prepare the aforementioned materials and to conduct the experiments described below were purchased from Sigma Aldrich unless indicated otherwise.

### Binding affinity measurements

The binding affinities of the Antp HD-DERT constructs were measured through fluorescence anisotropy-based protein titration experiments using a TAMRA-labeled 15-bp DNA duplex and various concentrations of proteins, as previously described (48). The affinity of the decoy 15-bp DNA was measured through fluorescence anisotropy-based competitive binding assays, as previously described (48). The apparent affinity of HMGB1 for the yeast tRNA mixture was measured through fluorescence-anisotropy-based competitive binding assays using 4 nM FAM-labeled DNA duplex containing a cisplatin-modification, 40 nM HMGB1, and various amounts of tRNA (10–10 000 nM). The apparent dissociation constant *K*_d_ for the HMGB1•tRNA complex was determined from the measured FAM fluorescence anisotropy data in conjunction with Equation (2) of (48) and *K*_d_ for the HMGB1 complex with cisplatin-modified-DNA (13).

### Measurements of target search kinetics in the presence of decoys

The target association kinetics in the presence of decoys were measured at 25°C using an Applied Photophysics SX20-LED stopped-flow spectrofluorometer. A polarized LED light with maximum intensity at 470 nm was used for excitation of the FAM fluorophore. The fluorescence anisotropy was measured in a real-time manner using two emission channels placed in a T-format configuration with a polarizer and a long-pass filter with a cutoff at 515 nm for each. All binding reactions were conducted under conditions of *T*_tot_ ≪ *P*_tot_ ≪ *D*_tot_, where *T*_tot_, *P*_tot_ and *D*_tot_ are the total concentrations of the target (i.e. probe), the protein, and the decoy, respectively. The apparent pseudo-first-order kinetic rate constant (*k*_app_) for target association was determined from the time course of fluorescence anisotropy through mono-exponential fitting. The rate constants *k*_app_ were measured at various concentrations of the proteins. For each kinetic rate constant, the measurement was replicated 8–10 times. MATLAB software (MathWorks) was used for nonlinear least-squares fitting. For the stopped-flow experiments on HMGB1 and its Δ30 variant, the following two solutions were rapidly mixed in a 1:1 volume ratio (80 μl each) by the stopped-flow device: a protein solution and a DNA/RNA solution of 10 nM FAM-labeled 20-bp DNA containing a cisplatin modification and 8000 nM tRNA as decoys. Both solutions were in a buffer containing 10 mM potassium phosphate (pH 7.5), 1 mM DTT, 1 mM MgCl_2_ and 100 mM KCl. Immediately after the flow for mixing had been stopped, the time course data of fluorescence anisotropy were collected for a period of 11 s with time intervals ranging from 0.02 to 0.05 s. For the stopped-flow experiments on the Antp HD derivatives, FAM-labeled 33-bp DNA containing an Antp recognition sequence (10 nM) was used as a fluorescence-labeled target, and a 15-bp DNA duplex (4000 nM) was used as a decoy. The sequence of the 33-bp DNA was FAM-AGCCATTACAGTGTACGCACGTACGGTGCACGA-3′, where the Antp recognition sequence is underlined. The sequence of the nonspecific 15-bp DNA was AGAAAGCAGACAGAG. The buffer was 10 mM potassium phosphate (pH 7.5) and 100 mM KCl. The processes of the association of the fluorescence-labeled target with the Antp-derivative proteins were analyzed through time-course data of the fluorescence anisotropy for a period of 10 s with time intervals of 0.001−0.05 s.

### NMR experiments

The NMR experiments were conducted using a Bruker Avance III 800-MHz NMR spectrometer equipped with a TCI cryogenic probe. To investigate electrostatic interactions of the C-terminal D/E repeats and the Antp HD, the ^1^H–^15^N TROSY spectra were recorded at 25°C for ∼0.1–0.2 mM ^15^N-labeled Antp HD-DERT11 and for the corresponding protein lacking DERT11 (Antp HD-MID) at 200, 300, 400, 500, 700 and 900 mM KCl. A coaxial NMR tube with the protein solution in a thinner inner tube and D_2_O in the outer tube was used to circumvent a problem in optimizing impedance matching of the cryoprobe ^1^H RF circuit due to the high ionic strengths (49). The protein solutions also contained 10 mM potassium phosphate (pH 7.5) and 1 mM 2,2-dimethyl-2-silapentane-5-sulfonate (DSS) for NMR chemical shift referencing (50). NMR spectra were processed and analyzed by NMR-Pipe (51) and NMRFAM-SPARKY (52) programs. Resonance assignment for the backbone NH groups in the homeodomain from a previous NMR study (53) on the Antp HD was used for the analysis. Using the approach described in our previous study (13), equilibrium constants *K_ai_* (= [X]/[P]) and the population of the autoinhibited state were determined from the salt-dependence data of the chemical shift difference (Δ*δ*) between the Antp HD-DERT11 and Antp HD-MID proteins.

### Computational modeling

#### All-atom simulations

To construct the 92-residue Antp HD-DERT protein, we used Model 1 (out of 20) of the NMR solution structure (PDB: 2HOA), whose core conformation was very similar to the crystal structures of the Antp HD bound to DNA and whose termini were extended. The NMR structure was used because it provided coordinates for all 60 residues of the Antp HD, whereas the X-ray crystal structures lack some N- and C-terminal residues. The p53 segment of the protein (15 residues) was taken from the NMR structure (PDB ID 2K8F), Chain B, using Model 1 (out of 10), where the segment was helical. The final 16 acidic residues were built in a helical conformation using PyMOL. The fragments were docked together using COOT. We used GROMACS (v. 2019.3) to run all-atom simulations of the free Antp HD-DERT protein and its complex with DNA. The force field parameters for the protein, SPC water and ions were derived from the AMBER99SB-ILDN force field. All structures were placed in a dodecahedral box, and solvated. Sodium and chloride ions were added to a concentration of 0.125M, with slight adjustments to neutralize the overall charge. All structures were subjected to minimization and NVT and NPT equilibration followed by six production runs of 3000 ns each.

#### Coarse-grained molecular dynamics simulations

The dynamics of the Antp HD-DERT derivatives and their binding to DNA were studied using coarse-grained molecular dynamics (CGMD) simulations. Each residue was represented by a single bead at the position of its Cα atom. The DNA was modeled with three beads per nucleotide, representing the phosphate, sugar, and base, and each bead was located at the geometric center of the group. The force-field applied in our simulations used a native-topology based model that includes a Lennard-Jones potential to reward native contacts and a repulsive potential to penalize non-native contacts (54–56). The DNA was modeled as a linear double-stranded B-DNA molecule with a length of 100 base pairs that remained in-place and rigid throughout the simulations. The positively charged residues of the protein (Lys, Arg) were assigned a point charge of (+1*e*) and the negatively charged residues (Asp, Glu) as well as the phosphate beads of the DNA backbone were assigned a negative charge of (–1*e*). The electrostatic potential between charged beads *q_i_, q_j_* was modeled by the Debye-Hückel interaction, which accounts for the ionic strength of a solute immersed in aqueous solution (57). The explicit form of the force field is reported elsewhere (58). The structure of the Antp HD was based on the conformation of the crystal structure PDB 9ANT. We have computational designed variants of Antp HD-DERT that vary in the number of negatively charged residues in the DERT. Mutating the DERT was achieved by keeping its length fixed but neutralizing charges located at its N-terminal. In the CGMD, the interactions between the D/E repeats and the other parts of the proteins were modeled by electrostatic interactions only. All other interactions within the proteins were modeled as repulsive (i.e. excluded volume), unless a contact was defined in the original structure using the CSU program (59). The dynamics of the proteins in isolation and in the presence of DNA were simulated using the Langevin equation (60). The dielectric constant was 80, and the salt concentration was varied as mentioned throughout the main text. For each system, we performed at least 80 simulations consisting of 2 × 10^8^ MD steps. Trajectory frames were saved every 1000 steps.

#### Rate equation-based simulations

The rate equations for the kinetic models are given in the Supplementary Data. The time courses of the concentrations of individual species were calculated by solving the rate equations numerically. The kinetic simulations were conducted with MATLAB scripts using the ‘ode15s’ stiff ordinary differential equation solver. The apparent rate constant *k*_app_ was determined by mono-exponential fitting to the simulated time course of the formation of the protein-target complex ([PT]/*T*_tot_). The equilibrium concentrations of individual species were calculated with MATLAB scripts using the ‘solve’ function by solving the simultaneous equations on the equilibrium constants and the mass conservation.

#### Statistical analysis

For kinetic measurements, at least 5 replicates were used to calculate the average values. Affinity measurements were triplicated. The reported experimental values are the averages and the standard errors of the means (SEMs). For the parameters from fittings, the error bars represent a confidence interval of 95%.

## RESULTS

### Autoinhibition by D/E repeats accelerates HMGB1–target association

**Figure 2.**
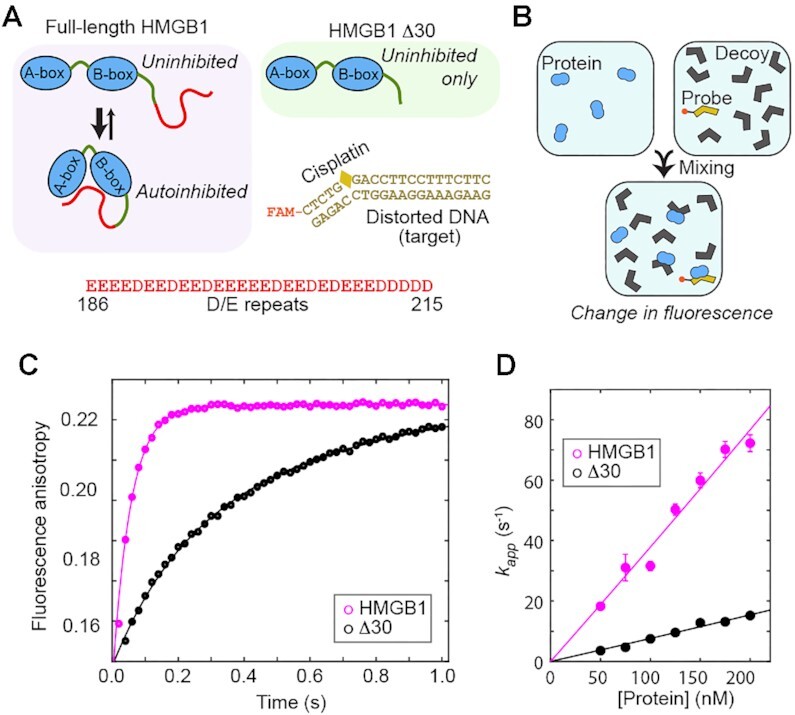
Autoinhibition of HMGB1 accelerates the HMGB1-target association in the presence of decoys. (**A**) Autoinhibition of HMGB1 occurs via electrostatic interactions of its D/E repeats with two DNA binding domains and other positively charged regions (8–10). Due to the lack of the D/E repeats, the Δ30 variant does not undergo autoinhibition. The fluorescence-labeled probe DNA modified by a cisplatin is also shown. (**B**) Stopped-flow fluorescence experiments for kinetic investigations of protein-target association in the presence of decoys. (**C**) Time-course of FAM fluorescence anisotropy upon mixing of a protein solution with a DNA solution containing the probe DNA and tRNAs as decoys. The concentrations of the protein, probe DNA and tRNAs were 50, 10, and 8000 nM, respectively. (**D**) Protein concentration dependence of the apparent pseudo-first-order rate constant *k_app_* for protein-target association in the presence of 8 μM tRNAs as decoys. The buffer was 10 mM potassium phosphate (pH 7.5), 1 mM DTT, 1 mM MgCl_2_ and 100 mM KCl.

HMGB1 is a multifunctional DNA-binding protein that undergoes dynamic autoinhibition. In the cell nuclei, HMGB1 binds to conformationally distorted DNA and acts as a DNA chaperone (40,43) HMGB1 exhibits strong affinities for various non-B-form DNA such as cisplatin-modified DNA, four-way junction DNA, and G-quadruplex (34,39,61). It can also bind to branched RNA (62). The C-terminal 30 residues of HMGB1 are D/E repeats (Figure 2A). This negatively charged segment causes dynamic autoinhibition through electrostatic fuzzy interactions with two DNA-binding domains and other positively charged regions within the same molecule (9,13). The equilibrium constant *K_ai_* for HMGB1 autoinhibition depends strongly on the salt concentration and is on the order of ∼10–10^2^ at physiological ionic strength (13).

To investigate the impact of D/E repeats on the target DNA search of HMGB1, the behavior of the full-length HMGB1 protein was compared with that of the Δ30 variant. Due to the lack of 30-residue D/E repeats mediated autoinhibition, the affinity of the Δ30 variant for the cisplatin-modified DNA is >∼100-fold stronger than that of the full-length protein at physiological ionic strength, as we previously demonstrated (13). Through stopped-flow fluorescence experiments, we measured the protein-target association kinetics for the full-length HMGB1 protein and the Δ30 variant in the presence of decoys (Figure 2B). FAM-labeled cisplatin-modified DNA (10 nM; 20 base pairs [bp]) was used as the target. tRNA (8000 nM) was used as a decoy. We chose tRNA rather than linear DNA duplex because branched RNA can effectively bind to HMGB1 (62) and RNA is highly abundant in the nuclei and may serve as decoys that trap HMGB1 *in vivo*. As shown in Supplementary Figure S1 in the Supplementary Data, the affinity of HMGB1 for tRNAs is ∼500-fold weaker than that for the cisplatin-modified DNA. In the stopped-flow experiments, we monitored the FAM fluorescence anisotropy after mixing a protein solution with a solution containing the probe DNA and tRNAs. From the time-course data, we determined the apparent rate constants for target association at various concentrations of the proteins in the presence of abundant decoys.

Some examples of the stopped-flow kinetic data are shown in Figure 2C. In these experiments, the full-length HMGB1 protein exhibited remarkably faster target association than the Δ30 variant, although autoinhibition makes the affinity of full-length HMGB1 weaker than that of the Δ30 variant. When the protein concentration was 50 nM, the target association of HMGB1 was 10 times as fast as that of the Δ30 variant lacking autoinhibition. The protein-concentration dependence of target association kinetics in the presence of decoys is generally nonlinear but expected to be virtually linear when the protein concentration is much lower than the decoy concentration (63). In fact, both proteins exhibited almost linear dependence on the protein concentration range tested (Figure 2D). At each concentration, the full-length HMGB1 protein exhibited a remarkably faster association than the Δ30 variant. These data suggest that autoinhibition via the D/E repeats accelerates the HMGB1–target association in the presence of decoys.

### Artificial autoinhibitory systems using D/E repeat tail

**Figure 3.**
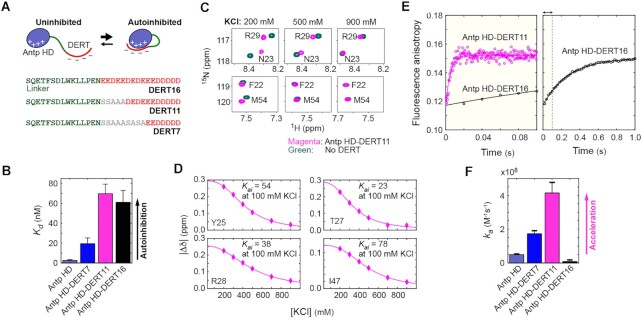
Artificial autoinhibitory systems using D/E repeat tail (DERT). (**A**) Protein constructs of the Antp homeodomain with a DERT (red) attached. The sequence shown in green is a linker adopted from p53 residues 15–29. (**B**) The dissociation constants (*K*_d_) for the complexes of the protein constructs with 15-bp DNA containing the Antp recognition sequence. (**C**) Overlaid heteronuclear ^1^H–^15^N correlation spectra recorded for Antp HD-DERT11 and the control protein with no DERT at various concentrations of KCl. Due to intra-molecular electrostatic interactions between HD and DERT, the NMR chemical shifts of the two constructs are significantly different at lower ionic strengths. (**D**) Chemical shift differences between Antp HD-DERT11 and the control protein with no DERT. The autoinhibition equilibrium constant *K_ai_* at 100 mM KCl was determined through the fitting to the KCl concentration dependence of chemical shifts for each residue, as previously described (13). The solid curves represent the best-fit curve. Variation in the *K_ai_* constant among different residues may reflect the dynamic nature of DERT in the autoinhibited state (13). (**E**) Stopped-flow fluorescence anisotropy data measured upon mixing 200 nM protein with a solution of 10 nM 33-bp FAM-labeled DNA (which contains an Antp recognition sequence) and 4 μM 15-bp decoy DNA at 100 mM KCl. Because Antp HD-DERT11 exhibited very fast kinetics, the time interval between anisotropy measurements was set to a smaller value for this protein. The larger noise is due to the shorter time interval. (**F**) Apparent association rate constant *k_a_* determined using the protein concentration dependence data from the stopped-flow fluorescence kinetics experiments.

To further examine whether D/E repeats cause acceleration of the target search kinetics, we made three protein constructs of the Antp homeodomain (HD; the overall charge, +12*e*) connected with D/E repeats through a linker (Figure 3A). The Antp HD is a DNA-binding domain that recognizes a TAATG sequence (64). In our Antp HD constructs, a D/E repeat tail (DERT) was attached, and we varied its length through substitutions to serine or alanine residues. The linker sequence between HD and DERT was adopted from the p53 residues 15–29, which are intrinsically disordered but have some (∼30%) helical propensity (65). Through fluorescence anisotropy-based assays, we measured the affinity for 15-bp DNA containing the Antp recognition sequence and confirmed that DERT caused autoinhibition for each protein construct (Figure 3B; see also Supplementary Figure S2 in Supplementary Data).

Through ^1^H–^15^N heteronuclear NMR experiments, we investigated the interactions between the HD and DERT regions. Our previous study showed that differences in NMR chemical shifts between the full-length HMGB1 protein and the Δ30 variant are strongly dependent on ionic strength due to the electrostatic nature of the autoinhibition via the D/E repeats (13). Likewise, NMR chemical shifts were considerably different between the constructs with and without DERT at low ionic strengths (Figure 3C). As the ionic strength was increased, the chemical shift differences decreased, approaching zero (Figure 3D). These results strongly suggest that DERT electrostatically interacts with the positively charged HD in the artificial autoinhibitory constructs. We analyzed apparent NMR chemical shifts, which reflect the autoinhibited and uninhibited states in fast exchange. As described previously (13), the ionic-strength dependence of the NMR chemical shift differences was fitted to a model function based on the counterion condensation theory. The autoinhibition equilibrium constant *K_ai_* for the Antp HD-DERT11 construct at 100 mM KCl from the fitting are shown in Figure 3D.

For each artificial autoinhibitory protein, we then performed stopped-flow fluorescence experiments to examine the impact of DERT on the target association kinetics in the presence of decoys. In this case, a FAM-labeled 33-bp DNA duplex containing an Antp recognition sequence was used as the target, whereas an unlabeled nonspecific 15-bp DNA duplex was used as a decoy. We monitored FAM fluorescence anisotropy in a real-time manner immediately after mixing a protein solution with a solution containing 0.01 μM target and 4 μM decoy. Examples of the time courses are shown in Figure 3E, and the apparent association rate constants determined from the protein concentration dependence are shown in Figure 3F.

Interestingly, the kinetic impact of autoinhibition on the protein-target association depended strongly on the DERT length. The Antp HD-DERT11 and -DERT7 constructs exhibited remarkable acceleration of the target association. The smaller effect for the DERT7 construct can be explained by a weaker autoinhibition as reflected in the *K*_d_ data. Although the DERT16 and DERT11 constructs exhibited a similar degree of autoinhibition in terms of affinity for the target, the Antp HD-DERT16 did not show any acceleration; rather, its target association was even slower than that of the original Antp HD (Figure 3F). Our further computational investigation and kinetic model will provide some insights into this different behavior of Antp HD-DERT16. Nonetheless, our current data on Antp HD-DERT clearly suggest that the autoinhibition-assisted acceleration of protein-target association can artificially be implemented through protein engineering.

### Target search process in coarse-grained molecular dynamics simulations

During target search process, proteins undergo sliding, hopping, and intersegment transfer processes in addition to dissociation and association with DNA (60,66,67). DNA-binding proteins with D/E repeats can adopt autoinhibited conformations when searching and change to uninhibited conformations at their target sites. Such a mechanism helps the DNA-binding proteins escape from decoys and locate their targets. Coarse-grained molecular dynamics (CGMD) simulations are suited for interpreting experimental data on conformational dynamics during the target DNA search process (68,69). Therefore, to gain further insight into how an autoinhibited protein can reach a target on DNA and make a transition into the uninhibited state, we conducted CGMD simulations for the Antp HD-DERT proteins.

We first applied the CGMD to characterize the autoinhibited state. The simulations showed that DERT dynamically interacts with the N-terminal tail, helix 3 (the DNA recognition helix), and the loop that connects helices 1 and 2. The conformational ensemble is broad due to high plasticity; thus, not all of the intramolecular interactions involving DERT may be found simultaneously in each structure. Similar structural features of the autoinhibited state identified in the CGMD simulations were also found in atomistic simulations (Supplementary Figure S3 in Supplementary Data), supporting the validity of CGMD. To address how the charge content of DERT affects DNA-binding kinetics, we used various constructs of Antp HD-DERT and computed the rates for their association with a target site modeled in the middle of linear 100-bp DNA in CGMD simulations. Other sites on the same DNA also electrostatically interact with the Antp HD and serve as decoys. The CGMD, thus, provides a useful tool for examining the role of autoinhibition in the target association kinetics at a resolution of a single DNA segment, which intrinsically includes a target and decoys.

**Figure 4.**
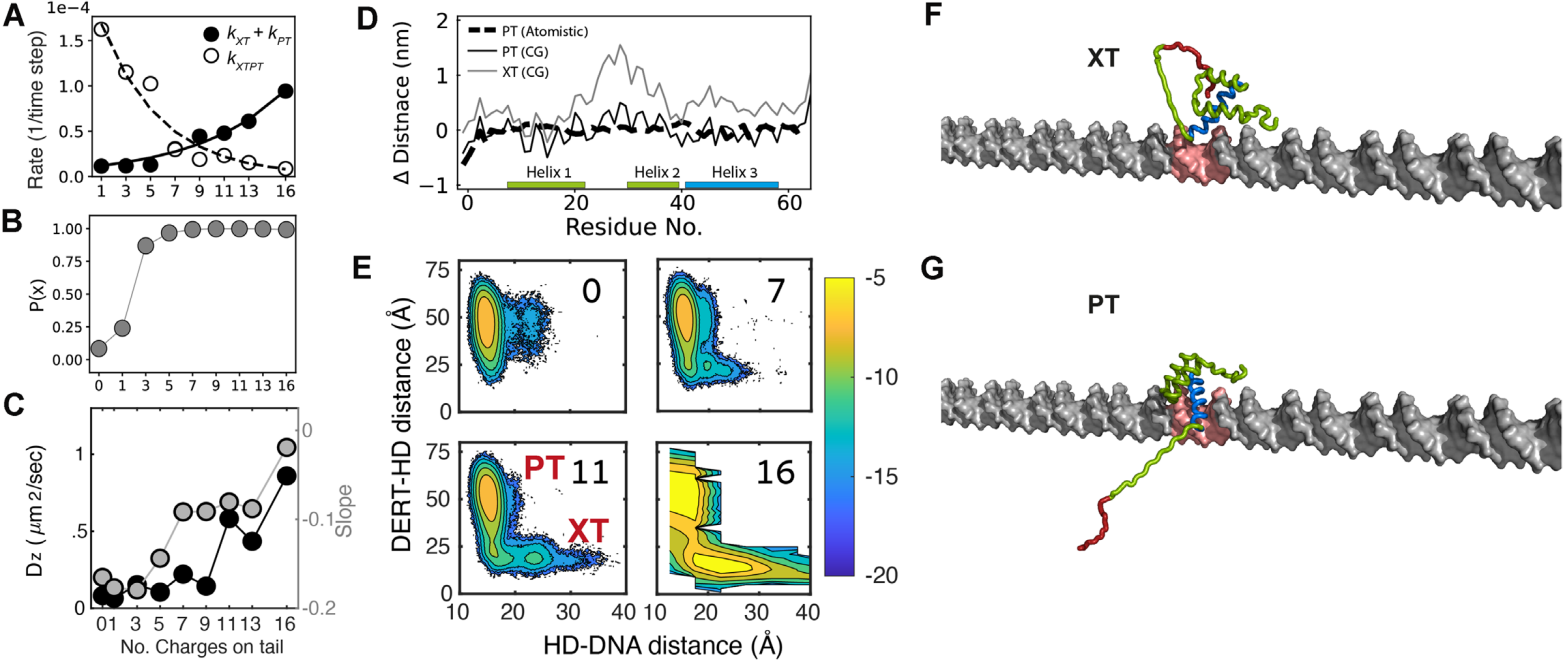
Computational analysis of DNA recognition by proteins that undergo dynamic autoinhibition via D/E repeats. (**A**) Kinetics of Antp HD-DERT computed from coarse-grained simulations. Black circles: the rate constant for initial binding of a protein molecule to its target on DNA. This binding event can occur as an autoinhibited (measured by *k*_XT_) or uninhibited (measured by *k*_PT_) state. White circles: the rate constant *k*_XTPT_ for the transition from the autoinhibited (XT) to uninhibited (PT) states while the protein is at the target. Rate constants are shown as a function of the number of negatively charged residues on the DERT. (**B**) The probability of Antp-HD DERT being in an autoinhibited state (state X) as a function of the number of charges on DERT. These data were obtained in the absence of DNA. (**C**) Left y-axis (black symbols): diffusion coefficient for linear diffusion along DNA as a function of number of charged residues on DERT. Right y-axis (gray symbols): the slope of the coupling between translation and rotation. A slope of -0.18 represents sliding.(83) Details of the diffusion coefficients and the slopes are described in Supplementary Data (see also Supplementary Figure S4). (**D**) The shortest distance between each Antp residue and the closest DNA phosphate is shown for the autoinhibited (XT, gray) and uninhibited states. (PT, calculated from CGMD (solid black line) and atomistic simulations (dashed black line)). The y-axis shows the difference from the distance in the crystal structure of Antp bound to DNA. (**E**) Two-dimensional maps showing the distance between DERT and Antp-HD (y-axis) vs the distance between Antp-HD and the DNA (x-axis). Each panel shows a map for a different variant, as indicated on the figure. Uninhibited (PT) and autoinhibited (XT) states are marked on the maps. The colors correspond to the population probability (in logarithmic scale) of conformations of Antp HD-DERT variants projected along the two-dimensional space. (**F**, **G**) Selected conformations of Antp HD-DERT at the target at either autoinhibited (Panel F, XT) or uninhibited (Panel G, PT) states. DERT is shown in red, and helix 3 is shown in blue. The target site, which is placed in the middle of linear 100-bp DNA, is highlighted in red. An example of the transitions from XT to PT is shown in Movie S1 in Supplementary Data.

Using the CGMD trajectories, we investigated the rate for the initial binding of the protein to the target. Note that the initial binding may not necessarily lead to the uninhibited complex with the target. Our data showed that the initial binding rate is larger for proteins with a larger number of negatively charged residues in DERT (Figure 4A, black circles). Because the population of the autoinhibited state increases upon an increase in the negative charge of DERT (Figure 4B), the faster initial binding can stem from faster diffusion in the autoinhibited state. Indeed, Figure 4C (left vertical axis) illustrates that the diffusion becomes faster upon an increase in the DERT charge content. Figure 4C (right vertical axis) also illustrates a shift from sliding to hopping modes as the DERT becomes more charged. Sliding is a 1-D diffusion of a protein molecule along DNA while maintaining contact with DNA. Hopping is a process involving dissociation, a short excursion of the free protein via 3-D diffusion, and reassociation with a proximal site on DNA. The shift to hopping can result in overall faster linear diffusion that may speed up the target search by the protein in the autoinhibited state.

Through statistical analysis of the mean passage time, we computed the rate constant *k_XTPT_* for switching from the autoinhibited complex to the uninhibited complex in the CGMD trajectories. The *k_XTPT_* data indicate that increasing the number of charged residues in DERT causes a slower transition (Figure 4A, white circles). Our computational results suggest that maximal acceleration of specific target recognition will be achieved when the two curves in Figure 4A intersect, that is, when there are ∼ 9 charged residues on HD-DERT. This trend closely resembles the experimental data shown in Figure 3F.

To gain further insight into how autoinhibition impact the target association kinetics, we calculated the average distance between each residue of the Antp HD and the nearest DNA phosphate in the simulations. The calculated distances for the uninhibited state are very close to the distances found in the crystal structure of Antp-HD bound to DNA in both atomistic and CGMD simulations (Figure 4D, dashed and solid black curve, respectively), but in the autoinhibited state helices 2 and 3 move ∼1 nm away from the DNA (Figure 4D, gray curve). The snapshots in Figure 4F–G clearly show that in the autoinhibited state, DERT binds helices 2 and 3, preventing HD from fully reaching its target. By contrast, in the uninhibited state, DERT is in an extended conformation, allowing helix 3 to mediate specific interactions with the target site. Movie S1 in the Supplementary Data shows a typical example of transitions from the autoinhibited complex to the uninhibited complex in CGMD trajectories. It appears that these transitions are facilitated by the electrostatic repulsive force between DNA and DERT, both of which are strongly negatively charged.

To better understand the transitions between the autoinhibited and uninhibited states at the target, we analyzed the energy landscape of the transitions observed in the simulations. Figure 4E shows the energy landscapes plotted using the distance between the center of mass (COM) of the DERT and the globular part of HD (y-axis), and the distance between the COM of HD and the DNA axis (x-axis). In the autoinhibited complex, one expects the distance between the DERT and HD to be short and the distance between the HD and DNA to be long, and vice versa for the uninhibited complex. Indeed, when the charge of DERT is small, the proteins mostly populate the uninhibited state, and autoinhibition is rare. As DERT becomes more charged, the population of the autoinhibited complex and the energy barrier between the autoinhibited and uninhibited complexes gradually increase, which is in accordance with the decrease in *k_XTPT_*.

### Kinetic model that explains the impact of D/E repeats

**Figure 5.**
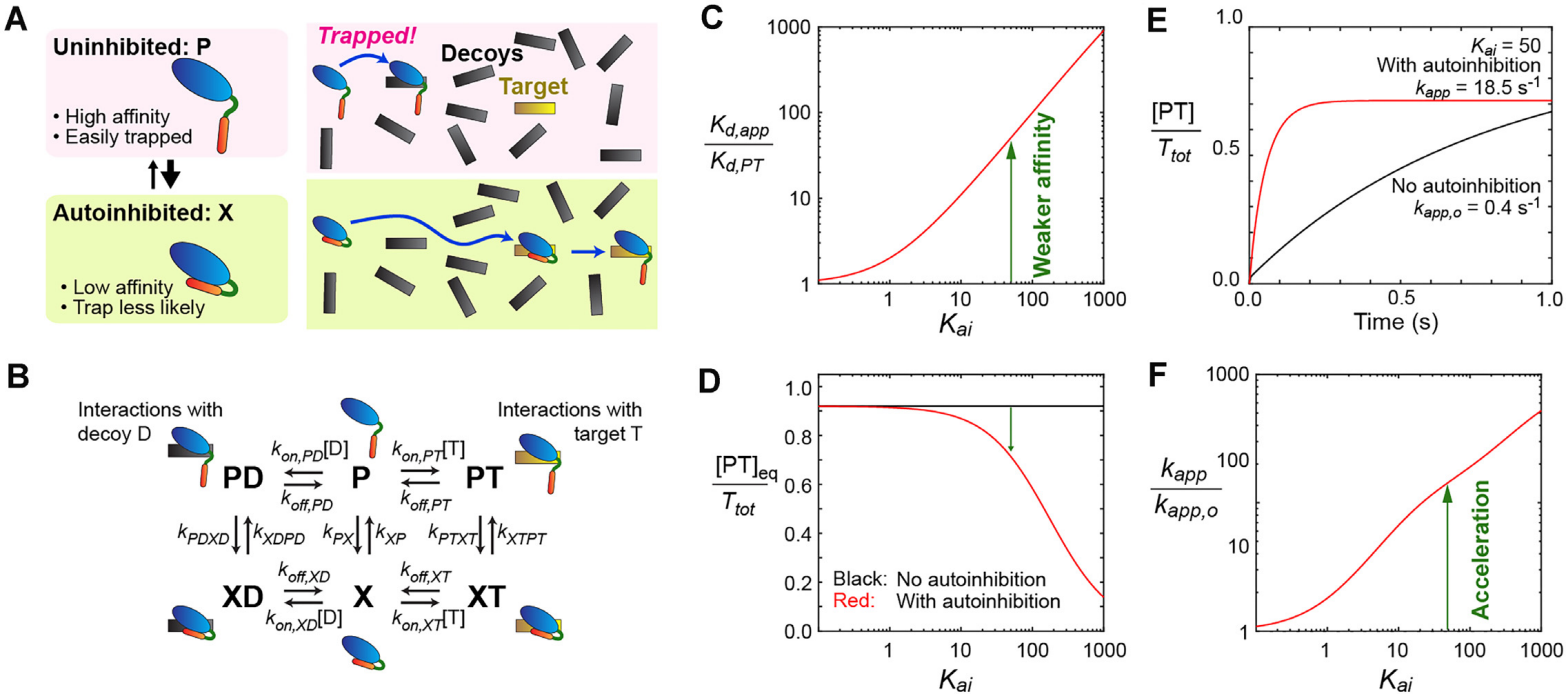
Kinetic model that explains how autoinhibition by D/E repeats can accelerate target search in the overwhelming presence of decoys. (**A**) Autoinhibition can reduce the risk of getting trapped by decoys. (**B**) A kinetic model of dynamic autoinhibition in the presence of targets and decoys. (**C**) Autoinhibition reduces the apparent affinity for the target. *K*_d,app_ represents the apparent dissociation constant for the protein-target complex in the absence of decoys. *K*_d,PT_ is the intrinsic dissociation constant for PT. *K_ai_* represents the equilibrium constant for autoinhibition (*K_ai_* = [X]_eq_/[P]_eq_). (**D**) Population of the protein-bound target in the presence of decoys. Note that the equilibrium populations of the protein-target complex (PT) in the autoinhibitory system may be only marginally smaller than in the system with no inhibition because autoinhibition also weakens the sequestration of the protein by decoys. (**E**) Time courses of the formation of the protein-target complex (PT) obtained by solving the rate equations. The initial conditions were [*D*] = *D*_tot_, [T] = *T*_tot_, [P] = *P*_tot_/(1 + *K_ai_*), [X] = *P*_tot_*K*_ai_/(1 + *K_ai_*), [PT] = [PD] = [XD] = [XT] = 0. The red curve is the results for the kinetic model shown in Panel B, whereas the black curve is the results for a system with no autoinhibition (i.e. X, XD and XT do not exist). The apparent pseudo-first-order rate constant *k_app_* for protein–target association is indicated for each case. (**F**) Kinetic impact of autoinhibition on protein–target association. This panel shows the ratio *k*_app_/*k*_app,o_, where *k*_app_ and *k*_app,o_ represent apparent pseudo-first-order rate constants for the formation of the protein–target complex (PT) in systems with and without autoinhibition, respectively. For Panels C–F, the following equilibrium and rate constants were used. The dissociation constants: *K*_d,PT_= 1 nM; *K*_d,PD_ = 500 nM; *K*_d,XT_ = 1 μM and *K*_d,XD_ = 500 μM. The intrinsic association rate constants: *k*_on,PD_= *k*_on,PT_ = *k*_on,XD_ = *k*_on,XT_ = 10^8^ M^−1^s^−1^. The rate constants for conformational transitions: *k*_XP_ = *k*_XTPT_ = *k*_XDPD_ = 10^3^ s^−1^; *k*_PX_ = *k*_XP_*K*_ai_; *k*_PTXT_ = *k*_XTPT_*K*_ai_*K*_d,PT_/*K*_d,XT_ and *k*_PDXD_ = *k*_XDPD_*K*_ai_*K*_d,PD_/*K*_d,XD_. The equations for *k*_PTXT_ and *k*_PDXD_ are based on the principle of detailed balance (84). For Panels D–F, *P*_tot_ = 200 nM, *T*_tot_ = 10 nM and *D*_tot_ = 8000 nM were used, where *P*_tot_, *T*_tot_ and *D*_tot_ represent the total concentrations of the protein, the target, and the decoy, respectively.

The acceleration of target search kinetics by D/E repeats can be explained using a kinetic model. A qualitative concept of the mechanism for the acceleration is depicted in Figure 5A. The free protein undergoes a dynamic equilibrium between the uninhibited (P) and autoinhibited (X) states. The uninhibited state allows for stronger binding to the target but exposes the protein to a high risk of getting trapped by decoys. By contrast, the autoinhibited state reduces the binding affinity but lowers the risk for the protein to get trapped by decoys. By having these two states in dynamic equilibrium, the protein can bind to the target more rapidly.

The kinetic model shown in Figure 5B offers more quantitative explanations. This model is akin to the model of Hammes *et al.* (70) for conformational selection (X→P→PT) and induced fit (X→XT→PT). The equilibrium constant *K_ai_* for autoinhibition is defined by *K_ai_* = [X]_eq_/[P]_eq_, where [ ]_eq_ represents an equilibrium concentration. *K_ai_* > 1 when the majority of the protein molecules are autoinhibited. The uninhibited protein (P) can form a stable complex (PT or PD) with a target (T) or a decoy (D), and the affinity for the target is substantially stronger, which means *K*_d,PT_ << *K*_d,PD_ in terms of the dissociation constant (*K*_d_). The autoinhibited protein (X) can weakly interact with the target or the decoy and form a transient complex (XT or XD), as seen in the CGMD simulations (Figure 4F). Furthermore, the electrostatic repulsion force expels the D/E repeats away from the DNA and induces the conformational transition to the uninhibited complex (Figure 4G; see also Movie S1).

The autoinhibition weakens the apparent affinity for the target, for which the apparent dissociation constant (*K*_d,app_) is given by *K*_d,PT_(1 + *K_ai_*)/(1 + *K*_ai_*K*_d,PT_/*K*_d,XT_), as explained in the Supplementary Data. Figure 5C shows this impact as a function of the equilibrium constant *K_ai_* for autoinhibition. For example, when *K_ai_*= 50, the autoinhibition weakens the apparent affinity by a factor of ∼50. One may expect that the autoinhibition causes a drastic decrease in the equilibrium population of the target-bound state. Interestingly, however, when the system involves an overwhelming number of decoys, the equilibrium population of the protein–target complex (PT) can be only marginally smaller in the autoinhibitory system than in the system with no autoinhibition (Figure 5D). This counterintuitive effect takes place because a less amount of the protein is trapped by decoys due to the reduced affinity.

Through simulations using this kinetic model, we examined how autoinhibition impacts the protein-target association kinetics in the presence of decoys. The rate constants involved in the model are indicated in the figure caption, and the rate equations are given in the Supplementary Data. The conditions were chosen to mimic the HMGB1 data shown in Figure 2C. In Figure 5E, the red curve shows the time-course data on protein-target association for the system involving autoinhibition in the presence of decoys, whereas the black curve shows the data for a corresponding system with no autoinhibition involved. These results resemble the experimental data shown in Figure 2C, suggesting faster target search kinetics in the presence of dynamic autoinhibition via D/E repeats. Figure 5F shows the extent of acceleration (i.e. the ratio of the apparent rate constants *k*_app_/*k*_app,o_ for target search in the presence and absence of dynamic autoinhibition) as a function of the *K_ai_* constant for autoinhibition. These data predict that the dynamic autoinhibition can greatly accelerate the protein-target association in the presence of decoys.

Our kinetic simulations suggest that autoinhibition can accelerate the protein-target association when (and only when) the induced-fit pathway is efficient. The fluxes of the conformational-selection (i.e. X→P→PT) and induced-fit (i.e. X→XT→PT) pathways are shown in Supplementary Figure S5 in Supplementary Data. In the system with decoy molecules (D), the conformational selection pathway is not efficient because the protein in the uninhibited state (P) can be easily trapped by decoys. Importantly, for the autoinhibition via D/E repeats, induced-fit can efficiently occur via electrostatic repulsion between the negatively charged IDR and DNA, as the CGMD simulations showed (Figure 4G and Movie S1)

Our further investigation reveals that the acceleration effect of autoinhibition is stronger when decoys are more abundant or exhibit higher affinity (Supplementary Figure S6 in Supplementary Data). As described in Supplementary Data, the acceleration effect can be approximated by the following:


(1) $$\begin{eqnarray*}\frac{{{k_{{\rm app}}}}}{{{k_{{\rm app,o}}}}} &\approx& {K_{ai}}\left( {\frac{{{k_{{\rm on,XT}}}}}{{{k_{{\rm on,PT}}}}}} \right)\ \frac{{{k_{{\rm XTPT}}}}}{{{k_{{\rm XTPT}}} + {k_{{\rm off,XT}}}}}\left( {\frac{{{P_{{\rm tot}}}}}{Q} + {K_{{\rm d,PT}}}} \right)\bigg/ \nonumber\\ && \left( {\frac{{{P_{{\rm tot}}}}}{{{Q_{\rm o}}}} + {K_{{\rm d,PT}}}} \right)\end{eqnarray*}$$

where *k*_on,XT_ and *k*_on,PT_ are the intrinsic association rate constants for the autoinhibited (X) and uninhibited (P) states, respectively; and *k*_XTPT_ is the rate constant for the transition from the autoinhibited complex (XT) to the uninhibited complex (PT) (see Figure 5B). *Q* and *Q*_o_ are given by Equations S26 and S28 in Supplementary Data and represent binding polynomials for quasi-equilibria on decoys. The derivation of Equation 1 and the validity range (Supplementary Figure S7) are given in Supplementary Data.

Equation (1) provides useful insights into the acceleration of protein-target association by autoinhibition. A condition necessary for the acceleration is that the intrinsic association rate of the autoinhibited protein (*k*_on,XT_) is sufficiently fast compared to the intrinsic association rate of the uninhibited protein (*k*_on,PT_). Otherwise, the acceleration effect will diminish due to *k*_on,XT_/*k*_on,PT_ << 1. Our CGMD simulations for Antp HD-DERT proteins show that the *k*_on,XT_/*k*_on,PT_ term in Equation (1) can be even larger than 1, which is well suited for the acceleration effect. Another necessary condition is that conformational transition from the autoinhibited complex (XT) to the uninhibited complex (PT) is sufficiently fast compared with the dissociation of XT. Otherwise, the acceleration will diminish due to *k*_XTPT_/(*k*_XTPT_ + *k*_off,XT_) << 1. The value of *k*_XTPT_/(*k*_XTPT_ + *k*_off,XT_) calculated from the CGMD changes from 0.9 for the DERT of 5 residues to 0.02 for the DERT with 16 residues. Thus, the CGMD data in conjunction with Equation 1 explain why there is an optimal length for the acceleration effect (Figure 3). Most likely, the slow target association of Antp HD-DERT16 is due to its slow conformational transition from XT to PT.

## DISCUSSION

Our current study demonstrates that dynamic autoinhibition via D/E repeats of appropriate length can accelerate the target DNA search process. This is radically different from the conventional notion of autoinhibition as a mechanism that creates an ‘off’ state in a molecular switch (21–23). The acceleration effect takes place because D/E repeats suppress association with decoys that impede the search process in systems involving numerous decoys. In other words, autoinhibition by D/E repeats allows the proteins to avoid distractions of decoys and thereby become able to capture the targets more rapidly.

Our coarse-grained simulations and kinetic model provide mechanistic insight into how D/E repeats can accelerate target search kinetics. The mechanism is similar to the concept of trade-off between search efficiency and binding affinity (68). However, reducing the affinity alone is insufficient for speeding up the search. A key requirement for the acceleration is that when encountering DNA, the protein must undergo an efficient transition from an autoinhibited conformation to an uninhibited conformation while interacting with DNA. The electrostatic repulsive force from DNA appears to induce the conformational change, pushing the negatively charged D/E repeats away from the DNA-binding domains, as seen in Movie S1. For autoinhibition to cause biologically meaningful acceleration of protein-target association, there is an optimal range of the equilibrium constant *K_ai_*. If autoinhibition is too weak, the acceleration effect will be too small due to trapping by the decoys. If autoinhibition is too strong, then the equilibrium population of the protein-target complex will be too low, diminishing the protein's function. For the system used for Figure 5D–F, when *K_ai_* is ∼10–10^2^, considerable acceleration can be achieved without severely reducing the equilibrium population of the protein-target complex. The *K_ai_* constants for HMGB1 (13) and the Antp HD-DERT11 protein fall into this range. For both systems, the affinity to DNA was decreased by about 100-fold by the autoinhibition via the D/E repeats which is coupled with an about 10-fold increase in binding rate.

Hundreds of proteins possess D/E repeats in each mammalian proteome (20). For example, 268 human proteins and 275 mouse proteins contain D/E repeats of 10 or more consecutive residues. However, only little is known about the functions of D/E repeats. Some D/E repeats have been demonstrated to undergo autoinhibition (5–13), others have been suggested to play a role in chaperone-like activities (14,71). For HMGB1, the role of D/E repeats in nucleosome remodeling was also proposed (72). Interestingly, ∼50% of all proteins containing D/E repeats are DNA/RNA-binding proteins (20). As demonstrated in our current study, these DNA/RNA-binding proteins may use D/E repeats to efficiently locate their targets in the genome containing billions of base pairs. For transcription factors that recognize specific sequences, there are millions of decoys in the mammalian genome, while functional targets are far fewer (30,31). Dynamic autoinhibition by D/E repeats can reduce the risk of sequestration by decoys. RNA-binding proteins likely face a similar situation (73) because RNA is even more abundant than DNA in cells. D/E repeats are more frequently found in higher eukaryotes (20). This may be relevant to the importance of D/E repeats as an accelerator of target search by DNA/RNA-binding proteins in higher eukaryotes, as a larger genome imposes more decoys.

Autoinhibition via D/E repeats appears to be well suited to accelerate the target search process. It remains to be examined whether other types of autoinhibition of DNA/RNA-binding proteins also accelerate the target search process. For example, autoinhibition of ADR2, C/EBPβ, Ets-1, ETV6, GCN2, Hfq, p53 and U2AF2 (25,26,28,74–80) are potentially interesting subjects of investigations in this regard. The acceleration requires the ability of the autoinhibited protein to form a transient complex (XT in the kinetic model shown in Figure 5B) that leads to an efficient induced-fit transition to the uninhibited complex with the target (PT). Autoinhibition via other IDRs might exhibit a behavior similar to D/E repeats.

Our study demonstrates the feasibility of protein engineering with D/E repeats. For a desired outcome, the length of D/E repeats and the linker may need to be optimized, as was the case for the Antp HD-DERT constructs (Figure 3). If the D/E repeat tail is too long, autoinhibition may be too strong and may not accelerate the target search process. If the D/E repeat tail is too short, the autoinhibition may be insufficient. The linker may also have to be optimized because its length and flexibility should affect the effective concentration of the inhibitory segment for the functional domain (81,82). Adding D/E repeats to a protein construct is relatively easy and may become a useful tool for protein engineering.

In conclusion, we have demonstrated that under certain conditions, dynamic autoinhibition can accelerate protein-target association in systems involving decoys. As observed for HMGB1, natural proteins containing D/E repeats may use this mechanism to efficiently associate with their targets in an environment involving numerous decoys. This mechanism can be implemented in other systems through protein engineering, as demonstrated for the Antp HD-DERT proteins. Such artificial autoinhibition via D/E repeats may become a useful tool to improve the kinetic properties of engineered/designed proteins. We have also gained insight into the conditions required for autoinhibition to accelerate target search. With D/E repeats of appropriate lengths suitable to satisfy these conditions, the proteins are able to avoid distractions of decoys and rapidly associate with the targets.

## DATA AVAILABILITY

All data are available in the paper and the Supplementary Data.

## Supplementary Material

gkad045_Supplemental_Files
