# Supplementary material for: Negatively charged, intrinsically disordered regions can accelerate target search by DNA-binding proteins
Source: Nucleic Acids Res. 2023 Feb 13;51(10):4701–12. doi: 10.1093/nar/gkad045 (PMC10250230; doi:10.1093/nar/gkad045)
Supplement: gkad045_Supplemental_Files [file gkad045_supplemental_files.zip › si113022a.pdf]

SUPPLEMENTARY DATA

Negatively charged, intrinsically disordered regions can accelerate target search by DNA-binding proteins

Xi Wang,[1] Lavi S. Bigman[2], Harry M. Greenblatt,[2] Binhan Yu,[1] Yaakov Levy,[2*] Junji Iwahara[1*]

[1] Department of Biochemistry and Molecular Biology, Sealy Center for Structural Biology and Molecular Biophysics, University of Texas Medical Branch, Galveston, Texas 77555-1068, USA

[2] Department of Chemical and Structural Biology, Weizmann Institute of Science, Rehovot 76100, Israel

*Corresponding authors. Emails: j.iwahara@utmb.edu and Koby.Levy@weizmann.ac.il

CONTENTS

1. **SUPPLEMENTARY TEXT**

1.1. Calculating rates and diffusion coefficients and rotation-translation coupling from coarse-grained molecular-dynamics simulations
1.2. Apparent dissociation constant for a protein that undergoes autoinhibition
1.3. The rate equations for the kinetic models in Figure 5
1.4. Flux analysis of the induced-fit and conformational-selection pathways
1.5. Analytical expression of the apparent rate constants $k_{app,o}$ and k_{app}

2. **SUPPLEMENTARY FIGURES**

Fig. S1. Competitive binding assay data for the HMGB1-tRNA interactions.
Fig. S2. Impact of D/E repeats tails (DERTs) on the DNA-binding affinity of the Antp HD.
Fig. S3. Structural characterization of the autoinhibited state of Antp-HD obtained from molecular dynamics simulations of free protein.
Fig. S4. Coupling between translation and rotation in one-dimensional diffusion of Antp-HD along DNA.
Fig. S5. Fluxes of the induced-fit (X→XT→PT) pathway and the conformational selection (X→P→PT) pathway.
Fig. **S6**. The acceleration effect of autoinhibition on protein-target association is stronger when the decoy is more abundant or exhibits higher affinity.
Fig. **S7**. Comparison of the approximate analytical expression of the rate constant kapp with the exact value from the numerical solutions of the rate equations.

3. **SUPPLEMENTARY MOVIE**

Movie S1. Transition from an autoinhibited complex to an uninhibited complex with DNA observed in the coarse-grained simulations for the Antp HD-DERT protein.

4. **SUPPLEMENTARY REFERENCES**

1. SUPPLEMENTARY TEXT

1.1. Calculating rates and diffusion coefficients and rotation-translation coupling from coarse-grained molecular-dynamics simulations

For the coarse-grained molecular dynamics simulations (CGMD) used to obtain the data shown in Figure 4, the specific binding of the Antp homeodomain (HD) to its target on DNA was modeled by defining a set of native-like contacts between the recognition helix (helix 3, residues 41-59) and the center of the DNA. The rates k_{XT} and k_{PT} shown in Figure 4A were determined on the basis of geometrical conditions and were calculated as the reciprocal of mean time for the center of mass (COM) of the protein to reach the target on the DNA. The rate k_{XTPT} for a transition from the autoinhibited state to the uninhibited state of the DNA-bound Antp HD-DERT was determined by calculating the reciprocal of mean time for the protein to form at least 50% of the possible contacts between helix 3 and the specific target site on DNA, once it was already positioned at the target site geometrically.

For Figure 4C, the mean square displacements (MSD) of the protein COM were calculated using the equation:

$$MSD(\tau) = \sum_{i=t_0}^{t-\tau} \frac{(r_{i+\tau}-r_i)^2}{t-\tau} = 2dD\tau \qquad \text{(s1)}$$

Where r is the position of the protein COM, t is the number of time steps measured, and τ is the measurement window ranging from t_0 to t. The slope of the MSD is $2dD$, where d is the dimensionality of diffusion and D is the diffusion coefficient. Unit conversion of diffusion coefficients was done as described in Ref. (1).

To investigate the coupling between translational and rotational movements of the protein around DNA (which was aligned along the z-axis), we analyzed correlation between the Z-coordinate of the protein COM and the angle θ of rotation calculated by:

$$\theta = \tan^{-1}\left(\frac{y}{x}\right) \qquad \text{(s2),}$$

where y and x are the corresponding coordinates of the protein COM. Examples of the Z-θ correlation plots are shown in Fig. S4. Sliding dynamics of proteins along DNA that exhibits coupling between rotation and translation shows a characteristic slope of θ when plotted versus translation along the z coordinate. As previously described (1), a slope of -0.18 is characteristics of sliding, whereas a greater slope may correspond to hopping dynamics.

1.2. Apparent dissociation constant for a protein that undergoes autoinhibition

When the system does not involve any decoys, the following partition function Z as a binding polynomial (2) can be defined for a protein that undergoes autoinhibition:

$$Z = 1 + K_{ai} + K_{d,PT}^{-1}[T] + K_{ai}K_{d,XT}^{-1}[T] \qquad \text{(s3)}$$

In this equation, the terms of 1, K_{ai}, $K_{d,PT}^{-1}[T]$, and $K_{ai}K_{d,PT}^{-1}[T]$ are for P, X, PT, and XT (see Figure 5B), respectively. Using this partition function, the population of the proteins bound to the target is given by:

$$p_{bound} = \frac{K_{d,PT}^{-1}[T]+K_{ai}K_{d,XT}^{-1}[T]}{Z} = \frac{\frac{K_{d,PT}^{-1}+K_{ai}K_{d,XT}^{-1}}{1+K_{ai}}[T]}{1+\frac{K_{d,PT}^{-1}+K_{ai}K_{d,XT}^{-1}}{1+K_{ai}}[T]} \qquad \text{(s4)}$$

For a protein that does not undergo autoinhibition, the corresponding partition function Z_o and the population $p_{bound,o}$ of the protein bound to the target are as follows:

$$Z_o = 1+K_{d,PT}^{-1}[T] \quad \text{(s5)}$$

$$p_{bound,o} = \frac{K_{d,PT}^{-1}[T]}{Z_o} = \frac{K_{d,PT}^{-1}[T]}{1+K_{d,PT}^{-1}[T]} \quad \text{(s6)}$$

Through comparison of Eqs. s4 and s6, one can obtain the apparent dissociation constant $K_{d,PT}^{app}$ for the complex of the protein that undergoes autoinhibition as follows:

$$K_{d,PT}^{app} = \frac{1+K_{ai}}{1+K_{ai}(\frac{K_{d,PT}}{K_{d,XT}})} K_{d,PT} \quad \text{(s7)}$$

1.3. The rate equations for the kinetic models in Figure 5

The rate equations for the kinetic model for the autoinhibitory system shown in Figure 5B are as follows.

$$\frac{d}{dt}[P] = -k_{on,PT}[P][T] - k_{on,PD}[P][D] - k_{PX}[P] + k_{off,PT}[PT] + k_{off,PD}[PD] + k_{XP}[X] \quad \text{(s8)}$$

$$\frac{d}{dt}[X] = -k_{on,XT}[X][T] - k_{on,XD}[X][D] - k_{XP}[X] + k_{off,XT}[XT] + k_{off,XD}[XD] + k_{PX}[P] \quad \text{(s9)}$$

$$\frac{d}{dt}[T] = -k_{on,PT}[P][T] - k_{on,XT}[X][T] + k_{off,PT}[PT] + k_{off,XT}[XT] \quad \text{(s10)}$$

$$\frac{d}{dt}[D] = -k_{on,PD}[P][D] - k_{on,XD}[X][D] + k_{off,PD}[PD] + k_{off,XD}[XD] \quad \text{(s11)}$$

$$\frac{d}{dt}[PT] = -k_{off,PT}[PT] - k_{PTXT}[PT] + k_{on,PT}[P][T] + k_{XTPT}[XT] \quad \text{(s12)}$$

$$\frac{d}{dt}[PD] = -k_{off,PD}[PD] - k_{PDXD}[PD] + k_{on,PD}[P][D] + k_{XDPD}[XD] \quad \text{(s13)}$$

$$\frac{d}{dt}[XT] = -k_{off,XT}[XT] - k_{XTPT}[XT] + k_{on,XT}[X][T] + k_{PTXT}[PT] \quad \text{(s14)}$$

$$\frac{d}{dt}[XD] = -k_{off,XD}[XD] - k_{XDPD}[XD] + k_{on,XD}[X][D] + k_{PDXD}[PD] \quad \text{(s15)}$$

The definitions of kinetic rate constants and molecular species are given in Figure 5B. These rate equations were used to simulate the time courses of protein-target association in the presence of the decoy.

For the model without any autoinhibited state (used for the black curve in Figure 5C), the following rate equations were used:

$$\frac{d}{dt}[P] = -k_{on,PT}[P][T] - k_{on,PD}[P][D] + k_{off,PT}[PT] + k_{off,PD}[PD] \quad \text{(s16)}$$

$$\frac{d}{dt}[T] = -k_{on,PT}[P][T] + k_{off,PT}[PT] \quad \text{(s17)}$$

$$\frac{d}{dt}[D] = -k_{on,PD}[P][D] + k_{off,PD}[PD] \quad \text{(s18)}$$

$$\frac{d}{dt}[PT] = -k_{off,PT}[PT] + k_{on,PT}[P][T] \quad \text{(s19)}$$

$$\frac{d}{dt}[PD] = -k_{off,PD}[PD] + k_{on,PD}[P][D] \quad \text{(s20)}$$

1.4. Flux analysis of the induced-fit and conformational-selection pathways

To investigate relative contributions of the induced-fit pathway (X→XT→PT) and the conformational-selection pathway (X→P→PT) in the autoinhibitory systems involving decoys, we

analyzed the fluxes of these pathways under various conditions. The flux through the induced-fit pathway (F_{IF}) and that through the conformational-selection pathway (F_{CS}) are as follows (3):

$$F_{IF} = \left(\frac{1}{k_{on,XT}[X][T]} + \frac{1}{k_{XTPT}[XT]}\right)^{-1} \quad \text{(s21)}$$

$$F_{CS} = \left(\frac{1}{k_{XP}[X]} + \frac{1}{k_{on,PT}[P][T]}\right)^{-1} \quad \text{(s22).}$$

To show the relative contribution of the flux through the induced-fit pathway, Fig. S5 displays $F_{IF}/(F_{IF} + F_{CS})$ and F_{IF}/F_{CS} for the system used for Fig. 5. The relative contribution of the induced-fit pathway clearly depends on the equilibrium constant K_{ai} for autoinhibition (Fig. S5A) but is virtually independent of the decoy concentration (Fig. S5B).

The dependence of the ratio F_{IF}/F_{CS} on the equilibrium constant K_{ai} (= $[X]_{eq}/[P]_{eq}$) can be explained as follows. The ratio F_{IF}/F_{CS} is given by:

$$\frac{F_{IF}}{F_{CS}} = K_{ai}\frac{k_{on,XT}}{k_{on,PT}}\frac{(k_{XP}[X]+k_{on,PT}[P][T])}{k_{XP}[X]}\frac{k_{XTPT}[XT]}{(k_{XTPT}[XT]+k_{on,XT}[X][T])} \quad \text{(s23)}$$

At the equilibrium, $k_{on,PT}$[P][T] = $k_{off,PT}$[PT] and $k_{on,XT}$[X][T] = $k_{off,XT}$[XT]. Due to the high stability of the uninhibited complex PT, it is likely that k_{XP}[X] >> $k_{off,PT}$[PT]. Therefore, the ratio F_{IF}/F_{CS} can be approximated by:

$$\frac{F_{IF}}{F_{CS}} \approx K_{ai}\left(\frac{k_{on,XT}}{k_{on,PT}}\right)\frac{k_{XTPT}}{k_{XTPT}+k_{off,XT}} \quad \text{(s24).}$$

Although [P], [X], [PT], and [XT] depend on the decoy concentration, the ratio F_{IF}/F_{CS} is virtually independent of the decoy concentration in the dynamic autoinhibitory system. When the conformational transition from XT to PT occur more rapidly than the dissociation of XT (i.e., k_{XTPT} >> $k_{off,XT}$), the flux through the induced-fit pathway (X→XT→PT) is predominant.

1.5. Analytical expression of the apparent rate constants $k_{app,o}$ and k_{app}

When the decoy concentration is much higher than the concentration of the other components, the protein-decoy association/dissociation processes reach quasi-equilibrium much more rapidly than the protein-target association/dissociation process (4). Taking advantage of the quasi-equilibrium, analytical expression of the rate constants $k_{app,o}$ and k_{app} can be obtained.

For the system with no autoinhibition, the apparent rate constant for the time course of [PT] is given by:

$$k_{app,o} = \frac{k_{on,PT}P_{tot}}{Q_o} + k_{off,PT} = k_{on,PT}\left(\frac{P_{tot}}{Q_o} + K_{d,PT}\right) \quad \text{(s25).}$$

The first and second terms represent the forward and backward processes (5). Q_o is a partition function for the protein at the quasi-equilibrium for the protein-decoy association/dissociation processes and is given by:

$$Q_o = 1 + K_{d,PD}^{-1}D_{tot} \quad \text{(s26).}$$

Strictly speaking, D_{tot} in Eq. s26 should be [D], the concentration of the decoy in the free state. However, in the current case, [D] ≈ D_{tot} because the decoy concentration is much higher than the concentrations of the protein and the target.

For the system with autoinhibition involving X, XD, and XT states, when the X→XT→PT pathway is the predominant association pathway and PT→XT→X is the predominant dissociation pathway, the apparent rate constant k_{app} can be approximated by:

$$k_{app} = \frac{k_{on,XT}K_{ai}P_{tot}}{Q}\frac{k_{XTPT}}{k_{XTPT}+k_{off,XT}} + k_{PTXT}\frac{k_{off,XT}}{k_{XTPT}+k_{off,XT}} \qquad \text{(s27)},$$

$$Q = 1 + K_{d,PD}^{-1}D_{tot} + K_{ai}(1 + K_{d,XD}^{-1}D_{tot}) \qquad \text{(s28)}.$$

Again, Q is a partition function for the protein at the quasi-equilibrium. However, unlike Q_o, this partition function Q involves not only the terms for P and PD, but also the terms for X and XD. Eq. s27 assumes the inequality $k_{XTPT} >> k_{PTXT}$, which makes the population of XT much smaller than that of PT. This condition is required for the analytical expression based on the steady-state approximation for systems involving a low-population intermediate (6). Using $k_{PTXT}/k_{XTPT} = K_{ai}K_{d,PT}/K_{d,XT}$ [which is based on the detailed balance principle (7)] along with $K_{d,XT} = k_{off,XT}/k_{on,XT}$, Eq. s27 can be transformed into:

$$k_{app} = K_{ai}k_{on,XT}\frac{k_{XTPT}}{k_{XTPT}+k_{off,XT}}\left(\frac{P_{tot}}{Q} + K_{d,PT}\right) \qquad \text{(s29)}$$

Although this is in a convenient form, Eq. 29 is applicable only when $K_{ai}K_{d,PT}/K_{d,XT} << 1$ (i.e., $k_{PTXT}/k_{XTPT} << 1$), which makes the steady-state approximation valid. In Fig. S7, this analytical expression is compared with the exact k_{app} rate constant from the time courses obtained through numerical solutions of the rate equations. Eq. 1 in the main text is obtained from Eqs. s25 and s29.

2. SUPPLEMENTARY FIGURES

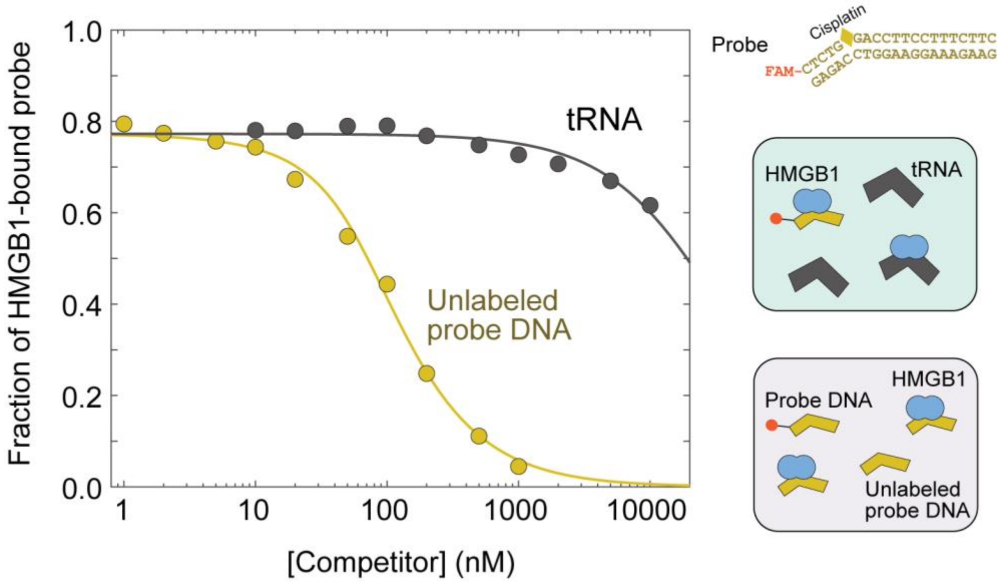

Fig. S1.

Competitive binding assay data for the HMGB1-tRNA interactions. In these assays, 40 nM HMGB1 was mixed with 4 nM FAM-labeled cisplatin-modified DNA and a competitor (tRNA or the unlabeled cisplatin-modified DNA) at varied concentrations. The buffer was 10 mM potassium phosphate (pH 7.5), 1 mM DTT, 1 mM $MgCl_2$, and 100 mM KCl. FAM fluorescence anisotropy was measured at 25˚C to obtain the fraction of the HMGB1-bound probe. The solid curves represent the best-fit curves obtained through nonlinear least-squares fitting with Eqs. 2-3 in Ref. (8). The apparent dissociation constants (K_d) determined from the fitting were $(8 \pm 2)\times10^3$ nM for tRNA and 17 ± 3 nM for the unlabeled cisplatin-modified DNA.

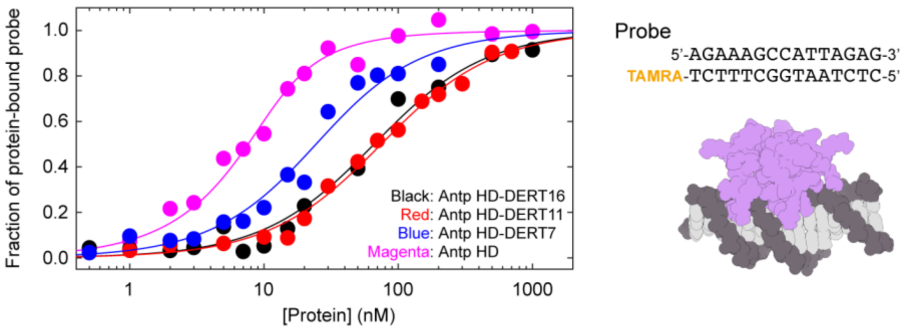

Fig. S2.

Impact of D/E repeats tails (DERTs) on the DNA-binding affinity of the Antp HD. The dissociation constants for the Antp HD protein constructs with and without DERT were measured through fluorescence anisotropy-based titration experiments. In these experiments, a TAMRA-labeled 15-bp DNA duplex (shown above) was used as a probe. 4 nM probe was mixed with each protein at various concentrations in the buffer of 20 mM potassium succinate (pH 5.8), 0.4 mM NaF, and 200 mM KCl. The fraction of protein-bound probe was obtained from TAMRA fluorescence anisotropy. The solid lines represent the best-fit curve. The dissociation constants (K_d) was determined to be 61 ± 12 nM for Antp HD-DERT16; 69 ± 10 nM for Antp HD-DERT11; 19 ± 6 nM for Antp HD-DERT7; and 2.6 ± 0.7 nM for Antp HD.

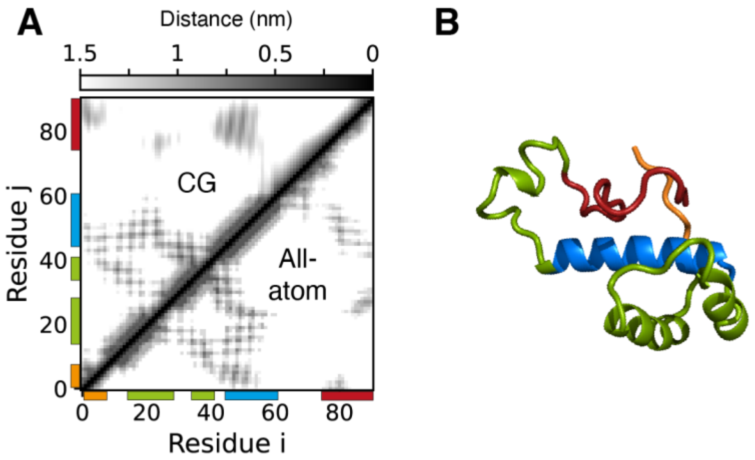

Fig. S3.

Structural characterization of the autoinhibited state of Antp-HD obtained from molecular dynamics simulations of free protein. (A) The ensemble of conformations is illustrated by an average contact map. Upper and lower diagonals of the map are from coarse-grained and atomistic simulations, respectively. The map shows the mean distance (in grayscale) between all pairs in the protein and exclude all distance longer than 1.5 nm. The structural elements of the protein are highlighted on the axes with the helices 1 and 2 shown in green, the recognition helix (helix 3) shown in blue, the N- terminal in orange and the D/E repeat in red. (B) A snapshot from atomistic simulations illustrating the D/E repeat interacting with helix 3 and the N-terminal tail.

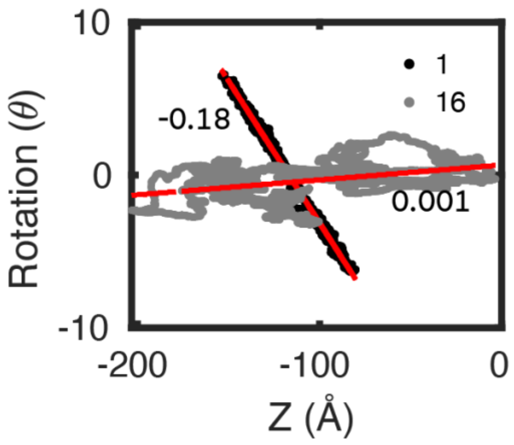

Fig. S4.
Coupling between translation and rotation in one-dimensional diffusion of Antp-HD along DNA. A coupled rotation-translation is a consequence of the protein following the DNA major groove. A complete rotation of 2π will therefore results in propagation of 31Å. Accordingly, a slope of 2 π /34=0.18 when plotting the protein rotation angle versus distance along the DNA axis (Z) is characteristic of coupled rotation-translation diffusion (i.e., sliding diffusion). A deviation from this value should be interpreted as weaker coupling between rotation and translation. Particularly a slope close to zero is characteristic of uncoupled rotation-translation diffusion (i.e., hopping diffusion). The slopes for DERT with 1 or 16 negative charges are shown and reflect that while sliding dominates the linear diffusion of Antp-HD with a DERT of a single negative charge, the diffusion of Antp-HD with a DERT of 16 negative charges follows hopping diffusion.

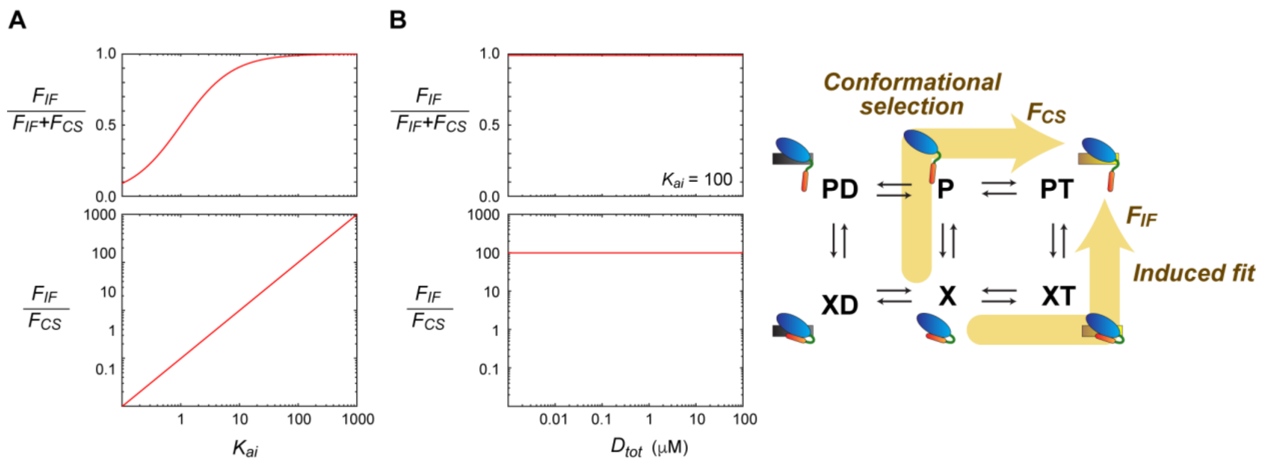

Fig. S5.

Fluxes of the induced-fit (X→XT→PT) pathway and the conformational selection (X→P→PT) pathway. (**A**) Relative magnitude of the flux through the induced-fit pathway as a function of the equilibrium constant K_{ai} for autoinhibition. The individual fluxes were calculated with Eqs. s21 and s22 along with the equilibrium concentrations of P, X, T, D, PT, PD, XT and XD. (**B**) Relative magnitude of the flux through the induced-fit pathway as a function of the total concentration of decoy (D_{tot}). For these panels, the following conditions were used: $K_{d,PT}$ = 0.1 nM; $K_{d,PD}$ = 10 nM; $K_{d,XT}$ = 1 μM; $K_{d,XD}$ = 100 μM; $k_{on,PD} = k_{on,PT} = k_{on,XD} = k_{on,XT} = 10^7$ $M^{-1}s^{-1}$; $k_{XP} = k_{XTPT} = k_{XDPD} = 10^3$ s^{-1}; $k_{PX} = k_{XP}K_{ai}$; $k_{PTXT} = k_{XTPT}K_{ai}K_{d,PT}/K_{d,XT}$ and $k_{PDXD} = k_{XDPD}K_{ai}K_{d,PD}/K_{d,XD}$. The equations for k_{PX}, k_{PTXT}, and k_{PDXD} are based on the principle of detailed balance (7). The total concentrations of the protein, the target, and the decoy (P_{tot}, T_{tot}, and D_{tot}, respectively) were set to 100 nM, 1 nM, and 5000 nM, respectively. These equilibrium constants, rate constants, and concentrations were identical to those used for Figure 5C-D.

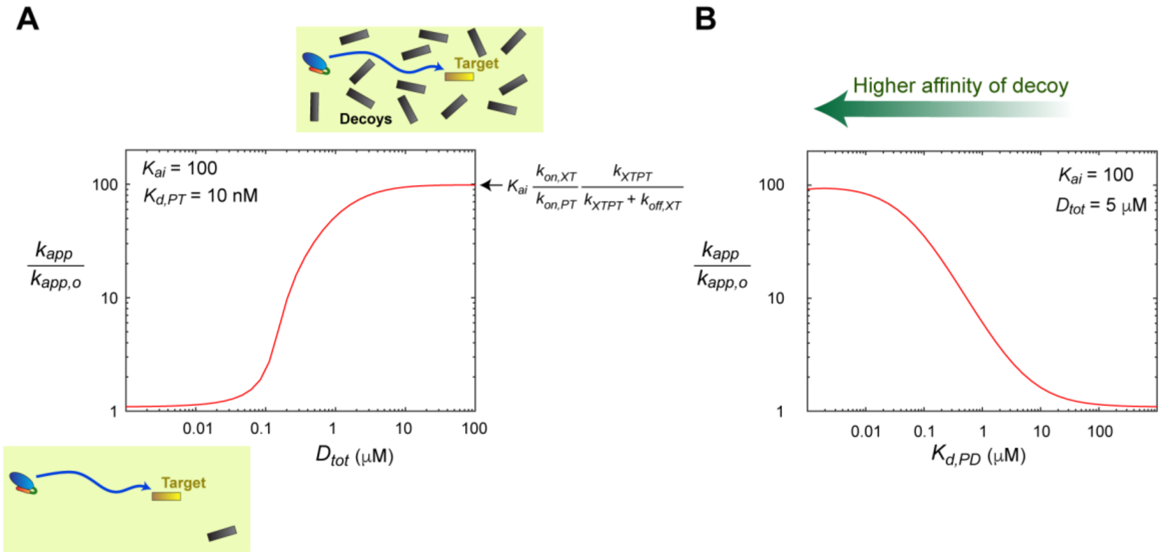

Fig. S6.

The acceleration effect of autoinhibition on protein-target association is stronger when the decoy is more abundant or exhibits higher affinity. (**A**) The acceleration effect $k_{app}/k_{app,o}$ as a function of the total concentrations of the decoy (D_{tot}). The conditions with K_{ai} = 100 and $K_{d,PD}$ = 10 nM were used for these simulations. (**B**) The acceleration effect $k_{app}/k_{app,o}$ as a function of the decoy affinity. D_{tot} = 5000 nM was used. For both panels, the following equilibrium and rate constants were used: $K_{d,PT}$ = 0.1 nM; $K_{d,XT}$ = 1 μM; $K_{d,XD}$ = 100 μM; $k_{on,PD} = k_{on,PT} = k_{on,XD} = k_{on,XT} = 10^7$ $M^{-1}s^{-1}$; $k_{XP} = k_{XTPT} = k_{XDPD} = 10^3$ s^{-1}; $k_{PX} = k_{XP}K_{ai}$; $k_{PTXT} = k_{XTPT}K_{ai}K_{d,PT}/K_{d,XT}$ and $k_{PDXD} = k_{XDPD}K_{ai}K_{d,PD}/K_{d,XD}$. The total concentrations of the protein and the target (P_{tot} and T_{tot}, respectively) were set to 100 nM and 1 nM, respectively.

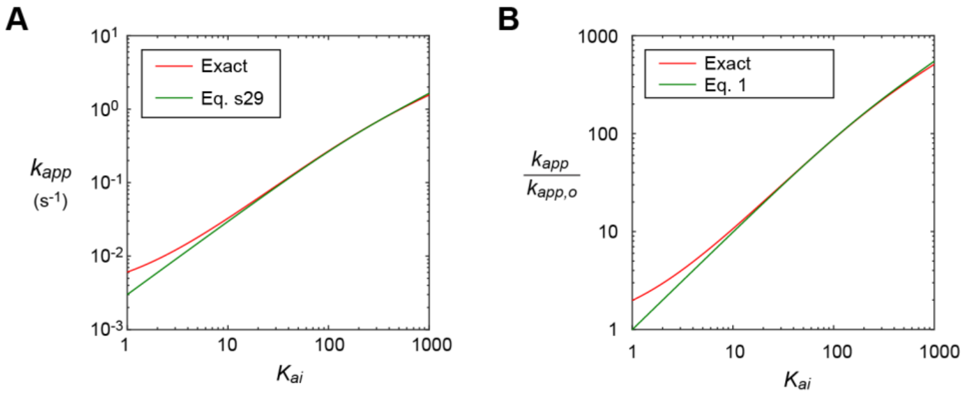

Fig. S7.

Comparison of the approximate analytical expression of the rate constant k_{app} with the exact value from the numerical solutions of the rate equations. (**A**) Exact k_{app} constant and approximation by Eq. s29. The approximation agrees well with the exact value when $K_{ai} >> 1$, which makes the induced-fit pathway predominant. (**B**) Exact $k_{app}/k_{app,o}$ ratio and approximations. The approximation by Eq. 1 agrees well with the exact ratio when $K_{ai} >> 1$.

3. SUPPLEMENTARY MOVIE

Movie S1.

Transition from an autoinhibited complex to an uninhibited complex with DNA observed in the coarse-grained simulations for the Antp HD-DERT protein. The position of the target is colored in red. See Figure 4F-G for snapshots of the autoinhibited and uninhibited complexes.

4. SUPPLEMENTARY REFERENCES

1. Bigman, L.S., Greenblatt, H.M. and Levy, Y. (2021) What Are the Molecular Requirements for Protein Sliding along DNA? *J Phys Chem B*, **125**, 3119-3131.
2. Barrick, D.E. (2018) *Biomolecular thermodynamics: from theory to application*. CRC Press, Boca Raton, FL.
3. Hammes, G.G., Chang, Y.-C. and Oas, T.G. (2009) Conformational selection or induced fit: A flux description of reaction mechanism. *Proc Natl Acad Sci U S A*, **106**, 13737.
4. Esadze, A. and Iwahara, J. (2014) Stopped-flow fluorescence kinetic study of protein sliding and intersegment transfer in the target DNA search process. *J Mol Biol*, **426**, 230-244.
5. Pollard, T.D. and De La Cruz, E.M. (2013) Take advantage of time in your experiments: a guide to simple, informative kinetics assays. *Mol Biol Cell*, **24**, 1103-1110.
6. Pilling, M.J. and Seakings, P.W. (1995) *Reaction kinetics*. Oxford University Press, Oxford.
7. Hammes, G.G. (2000) *Thermodynamics and kinetics for the biological sciences*. 3 ed. Wiley-Interscience, New York.
8. Zandarashvili, L., Nguyen, D., Anderson, K.M., White, M.A., Gorenstein, D.G. and Iwahara, J. (2015) Entropic Enhancement of Protein-DNA Affinity by Oxygen-to-Sulfur Substitution in DNA Phosphate. *Biophys J*, **109**, 1026-1037.
